# Prevalence of *Toxocara* infection and associated risk factors: a cross-sectional study in Zhejiang, China

**DOI:** 10.1186/s40249-025-01312-w

**Published:** 2025-06-05

**Authors:** Yi Yang, Die Zhong, Haiyan Wu, Zhiwei Xiong, Chenyu Yang, Lei Yang, Hongli Zhang, Beibei Wu, Guangxu Ma

**Affiliations:** 1 College of Animal Sciences, Zhejiang Provincial Key Laboratory of Preventive Veterinary Medicine, Zhejiang University, Hangzhou, China; 2 ZJU-Xinchang Joint Innovation Centre (TianMu Laboratory), Gaochuang Hi-Tech Park, Xinchang, China; 3 Zhejiang Key Lab of Vaccine, Infectious Disease Prevention and Control, Zhejiang Provincial Center for Disease Control and Prevention, Hangzhou, 310015 China; 4 Zhejiang Provincial Center for Animal Disease Control and Prevention, Hangzhou, 311199 China

**Keywords:** Toxocariasis, Dog, Cat, Human, Prevalence, Risk factor, Model zone of common prosperity, One Health

## Abstract

**Background:**

Human toxocariasis, caused by the zoonotic parasites *Toxocara canis* (dog roundworm) and *T. cati* (cat roundworm), affects approximately 19% of the global population, ranking it among the most prevalent neglected infection of poverty. However, public awareness about this zoonotic disease has not yet been achieved in China. In this study, we conducted an epidemiological survey to assess the prevalence and risk factors of *Toxocara* infection in dogs and cats, as well as toxocariasis or *Toxocara* exposure in humans in Zhejiang.

**Methods:**

An epidemiological survey was conducted between January 2023 and April 2024 to ascertain the prevalence of *Toxocara* infection in Zhejiang, where has been set to be a model for common prosperity in China. Fecal samples from dogs (*n* = 1156) and cats (*n* = 818) were examined for *Toxocara* eggs using the saturated saline floatation method and molecular tools, while human serum samples (*n* = 347) were tested for antibodies against *Toxocara* species by an enzyme-linked immunosorbent assay. Risk factors for *Toxocara* infection in dogs, cats and humans were analyzed using logistic regression models.

**Results:**

The overall prevalence of *Toxocara* infection was 5.36% in dogs, 2.08% in cats, and 12.10% in humans in Zhejiang, China. Age (≤ 6 months, *OR* = 6.22, *P* = 0.026), season (autumn, *OR* = 13.93, *P* = 0.017 and spring, *OR* = 11.07, *P* = 0.027) and deworming frequency (< 4 times/year, *OR* = 0.18, *P* < 0.001) were identified as major risk factors for *T. canis* infection in dogs, whereas residing in an animal shelter (*OR* = 13.14, *P* = 0.020) was a risk factor for *T. cati* infection in cats. Occupation exposure (*OR* = 4.53, *P* = 0.009) was the most significant risk factor for *Toxocara* infection in humans.

**Conclusions:**

Due to the good economic status and social welfare, the prevalence of *Toxocara* infection in dogs, cats and humans is relatively low in Zhejiang, China. However, an “One Health” paradigm about human toxocariasis intervention is lacking and the risk factors (particularly pet deworming and occupational exposure) for *Toxocara* infection and transmission warrant improved public awareness.

**Graphical Abstract:**

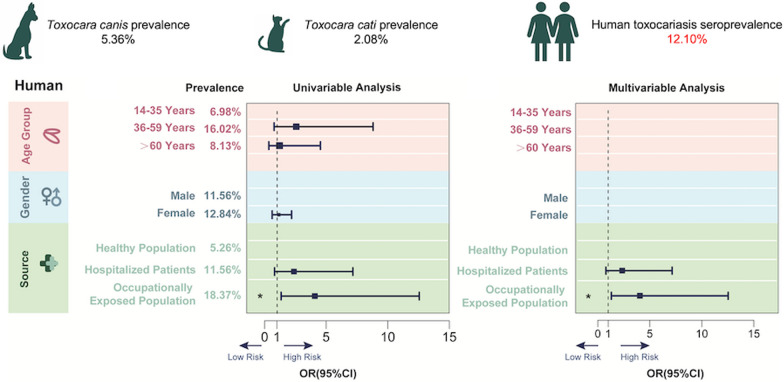

**Supplementary Information:**

The online version contains supplementary material available at 10.1186/s40249-025-01312-w.

## Background

Human toxocariasis is a neglected parasitic disease of poverty mainly caused by the dog roundworm *Toxocara canis* and, to a lesser extent, by the cat roundworm *Toxocara cati* [1, 2]. It has been estimated that the global prevalence of *Toxocara* infection is 11% in dogs, 17% in cats, and 19% in humans [3, 4]. Although most people infected with *Toxocara* species are asymptomatic, a series of clinical syndromes including visceral larva migrans (VLM), ocular larva migrans (OLM), neural toxocariasis (NT), and covert toxocariasis have been well documented [1, 5–7]. Associations of *Toxocara* infection in humans with allergic disorders (e.g., pruritus and asthma) and with delayed cognitive development even neural degenerative diseases (e.g., epilepsy, idiopathic Parkinson’s disease and Alzheimer’s disease) also have been proposed [8–10]. Keeping and close contacting with cats and dogs, and bad hygiene situations or habits are the main risk factors for *Toxocara* infection in China [11]. Clearly, a “One Health” concept integrating epidemiology, diagnosis, and treatment should be preferably established globally to enhance our understanding and the control of human toxocariasis [12, 13].

As the source of infection, canines (e.g., dogs, foxes and wolves) are the definitive host of *T. canis* while felines (e.g., cats) are the final host of *T. cati* [14–16]*.* Within the intestine of these host animals, adult females of *T. canis* or *T. cati* mate with adult males then excrete fertilized eggs into the feces. The eggs become larvated and infectious (also known as infective eggs) in the environment, which are resistant and capable of host infection for months. Accidental ingestion of soil, water or food containing these infective eggs lead to a *Toxocara* infection in canines/felines, and a range of other animals including humans serving as paratenic hosts [17]. Within these animals, larvae released from the infective eggs penetrate the intestinal wall, enter the bloodstream, undergo visceral migration from the liver to lungs (i.e., hepato-pulmonary phase), disperse from the tracheal to the musculature and brain (i.e., myotropic-neurotropic phase), causing VLM, NT and OLM in humans [1, 5, 18], then are developmentally arrested. In dogs and cats, the developmentally arrested larvae can be activated during late pregnancy and lactation of dogs/cats, which migrate to the placenta and mammary glands, then transmit to and mature in the intestine of puppies/kittens [16–20]. Although the activation of arrested larvae of *Toxocara* species also has been reported in paratenic hosts (e.g., mouse), little is known about the outcomes, particularly in humans [21–23]. Beyond the biology of *Toxocara* species, public concerns of toxocariasis are about whether their dogs and cats are infected, if so, the key question is how to prevent the transmission to humans.

The diagnosis of *Toxocara* infection in dogs and cats is simple and easy, essentially based on the identification of roundworms in the feces or microscopic detection of characterized eggs in the feces [24, 25]. By contrast, accurate diagnosis of toxocariasis in humans is much more difficult, mainly because there are no developed adults excreting eggs into the feces of people that are infected with *Toxocara* species [1, 17]. Serologic tests, such as immunoblot and enzyme-linked immunosorbent assay (ELISA), have been employed for detecting IgG-specific antibodies against the excretory/excretory antigens of *Toxocara* (TES antigens) [26]. Molecular tools, such as polymerase chain reaction (PCR), have been developed for the detection of *Toxocara* DNA fragment in the feces of dogs/cats and for the amplification of circulating microRNAs in the serum or cerebrospinal fluid of host animals [27, 28], though further improvements in terms of cost and efficiency are still warranted. Nonetheless, the availability of these tools enables surveillance of *Toxocara* infection in dogs and cats as well as toxocariasis in humans.

In this study, epidemiological survey and studies were conducted to ascertain the prevalence and risk factors of *Toxocara* infection in dogs and cats, as well as toxocariasis or *Toxocara* exposure in humans in Zhejiang, the first demonstration zone for common prosperity of China.

## Methods

### Study area

An epidemiological study on *Toxocara* infection in dogs and cats was conducted across several cities in Zhejiang Province, China, including Hangzhou, Huzhou, Jinhua, Ningbo, Jiaxing, Lishui, Quzhou, Shaoxing, Taizhou, Wenzhou, and Zhoushan. Additionally, a study on human toxocariasis was carried out in Hangzhou, Ningbo, Taizhou, and Zhoushan within the same province.

### Study population

The study included dogs and cats from pet hospitals, animal shelters, pet stores, and residential communities, as well as adult individuals who enrolled in psittacosis surveillance by the Zhejiang Provincial Center for Disease Control and Prevention (Zhejiang CDC).

### Sample size determination

Based on the estimated prevalence of *Toxocara* infection in dogs, cats and humans in China [4, 11, 29–31], the minimum sample sizes were calculated using the WinEpi software (http://www.winepi.net/uk/index.htm). The required minimum sample sizes were 295 for dogs, 187 for cats, and 237 for humans.

### Sample collection and processing

A total of 1156 fresh fecal samples were collected from dogs and 818 fresh fecal samples from cats between March 2023 and April 2024 to meet the minimum sample size requirement. Each sample collected from an animal was packed into a clean plastic bag (Shanghai Zhonghe Chemical Technology Co., LTD, Shanghai, China), and relevant information (i.e., age, gender, breed, source, and deworming frequency) was recorded. The fecal samples were stored at 4 °C until they were microscopically examined and/or processed for DNA extraction within one week.

A total of 347 serum samples were collected between January 2023 and October 2023, and stored at −80 °C at the Zhejiang CDC. The curation of information (e.g., age, gender, healthy status, and occupation), as well as serum processing and examination, were conducted in the Key Laboratory of Microbiology, Zhejiang CDC.

### Microscopic identification

Each fecal sample (about 5 g) was crushed, mixed with 50 ml of purified water, and filtered through a 100-mesh sieve. The filtered liquid was centrifuged at 3000 × *g* for 10 min, after which the precipitation was resuspended with 50 ml of saturated saline and subjected to centrifugation at 3000 × *g* for 10 min. A metal ring (0.5 cm in diameter) was used to touch the surface of supernatant and transfer liquid drops onto a slide. After placing with a coverslip, the slide was examined for eggs of parasitic worms under a compound microscope (Olympus, Nagano prefecture, Japan). At least three slides were prepared and examined by two technicians for each fecal sample.

### Molecular identification

Genomic DNA was extracted from fecal sample that were microscopically identified as containing eggs of *Toxocara* and related species, using the DNeasy PowerSoil Pro Kit (Qiagen, Hilden, Germany) in accordance with the manufacturer’s instructions. Using the extracted genomic DNA as template, sequence of the internal transcribed spacer region 2 (ITS-2) was amplified using a well-established polymerase chain reaction (PCR) method [32]. Primers used for *T. canis*, *T. cati*, and *To. leonina* identification were Tcan1: 5′-AGTATGATGGGCGCGCCAAT-3′, Tcat1: 5′-GGAGAAGTAAGATCGTGGCACGCGT-3′, and Tleo1: 5′-CGAACGCTCATATAACGGCATACTC-3′, respectively, as well as the reverse NC2: 5′-TTAGTTTCTTTTCCTCCGCT-3′. The reaction mixture was composed of 10 µl 2 × *Taq* Master PCR Mix (E005-01B, Novoprotein), 2.0 µl primers (10 pmol/µl; Zhejiang Shangya Biotechnology Co., LTD), 1.0 µl template DNA and 7 µl nuclease-free water. The thermal conditions for *T. canis* comprised one cycle of 94 °C for 1.5 min, followed by 35 cycles of 94 °C for 20 s, 56 °C for 25 s, and 72 °C for 30 s, with a final extension of 72 °C for 5 min on a thermal cycler (ProFlex PCR instrument, Applied Biosystems, Foster, ABI). The thermal conditions for *T. cati* comprised one cycle of 94 °C for 1.5 min, followed by 35 cycles of 94 °C for 20 s, 57 °C for 20 s, and 72 °C for 25 s, with a final extension of 72 °C for 5 min. The thermal conditions for *To. leonina* comprised one cycle of 94 °C for 1.5 min, followed by 35 cycles of 94 °C for 20 s, 54 °C for 20 s, and 72 °C for 30 s, with a final extension of 72 °C for 5 min. PCR products were subjected to electrophoresis on a 1% agarose gel and subsequently sequencing. The obtained sequences were searched against the GenBank NR database for species identification.

### Enzyme linked immunosorbent assay (ELISA)

Human serum samples were thawed on ice, then inactivated at 56 °C in a water bath for 30 min. An immunoglobulin G indirect ELISA kit (NovaTec Immundiagnostica GmbH, Berlin, Germany) was used to detect the presence of anti-*Toxocara* antibodies in these serum samples according to the manufacturer’s instructions. All serum samples were 1∶100 diluted and spiked into the wells of 96-well plate. All samples were processed in triplicate, with the negative, positive, and critical quality controls included. The absorbance of each well was measured at 450 and 620 nm using an enzyme labeling instrument (BIO-RAD model680, USA). Data for each well was obtained by subtracting the absorbance at 450 nm from the absorbance at 620 nm.

All reactions should meet the run validation criteria according to the manufacturer’s instructions, with absorbance value of substrate blank < 0.100, negative quality control < 0.200 and < critical value, positive quality control> critical value. The critical value of substrate blank was 0.150–1.300. Optical density reading for each well was subtracted the value of substrate blank, then used for the calculation of mean absorbance value for each sample. The mean value was then multiplied by 10 for the determination of negative or positive results according to the biological activity units outlined by the manufacturer. The value ≥ 11 activity units was considered as positive, the value ≥ 9 activity units and < 11 activity units was considered as ambiguous, and the value < 9 activity units was considered as negative.

### Statistical analysis

Data was presented as mean with 95% confidence intervals (95% *CI*). The prevalence and binomial 95% *CI* were calculated using the epidemiologic calculator EpiTools software (Ausvet, Fremantle, Australia). Logistic regression modeling was performed to explore risk factors including source, age, sampling time and deworming frequency for *Toxocara* infection in dogs and cats, using the *glm* function in R environment v.4.4.1 (Lucent Technologies, Jasmine Mountain, USA). “pet store”, “> 6 months of age”, “summer”, and “no deworming” were used as references for source, age, sampling time, and deworming frequency, respectively, in binary multifactorial logistic regression analyses. All variables were initially subjected to univariable analysis with a threshold of *P* value < 0.05, and then to a multivariate analysis. In the risk factor analysis for human toxocariasis, age was categorized into three age groups: young (14–35 years), middle-aged (36–59 years), and old (≥ 60 years). The model was based on the terms “14–35 years”, “male”, and “healthy population” as the reference for age group, gender, and source, respectively, for binary multifactor logistic regression analysis. A statistical significance level of 5% was used (*P* value < 0.05). An advantage ratio (odds ratio, *OR*) value was deemed statistically significant if the 95% *CI* did not encompass a value of 1.

## Results

### Prevalence of *Toxocara* infection in dogs in Zhejiang

Based on microscopic detection and molecular identification, eggs of *T. canis* were screened in 1156 fecal samples collected from dogs in Zhejiang, with a prevalence of infection calculated to be 5.36% (62/1156; 95% *CI*: 4.21–6.82; Table 1; Additional information 1: Table S1). Apart from *T. canis*, *To. leonina*, Ancylostomatidae (hookworm), and *Trichuris vulpis* (whipworm) were also detected in these fecal samples, with prevalences of 0.17% (2/1156), 7.61% (88/1156), and 1.56% (18/1156), respectively (Table 1). In certain fecal samples, eggs of both *T. canis* and Ancylostomatidae, *T. canis* and *Tr. vulpis*, or Ancylostomatidae and *Tr. vulpis* were identified, indicating co-infection rates of 0.35% (4/1156), 0.09% (1/1156), and 0.61% (7/1156) for these nematodes in dogs, respectively.

**Table 1:** Prevalence of *Toxocara* and related nematode infection in dogs and cats in Zhejiang, China

Regions	Prevalence of nematode infection in dogs, % (*n*/*n*)				Prevalence of nematode infection in cats, % (*n*/*n*)			
	*Toxocara canis*	Ancylostomatidae	*Toxascaris leonina*	*Trichuris vulpis*	*Toxocara cati*	*Toxocara canis*	*Toxascaris leonina*	*Trichuris campanula*
Hangzhou City	3.72 (11/296) 95% *CI*: 2.09–6.53	11.49 (34/296) 95% *CI*: 8.34–11.49	0.34 (1/296) 95% *CI*: 0.02–1.89	3.38 (10/296) 95% *CI*: 1.85–6.11	1.54 (8/519) 95%* CI*: 0.78–3.01	0.00 (0/519) 95% *CI*: 0.00–0.73	0.58 (3/519) 95% *CI*: 0.16–1.69	0.00 (0/519) 95% *CI*: 0.00–0.73
Huzhou City	0.00 (0/124) 95% *CI*: 0.00–3.01	17.74 (22/124) 95% *CI*: 12.02–25.40	0.00 (0/124) 95% *CI*: 0.00–3.01	0.00 (0/124) 95% *CI*: 0.00–3.01	0.00 (0/48) 95% *CI*: 0.00–7.41	0.00 (0/48) 95% *CI*: 0.00–7.41	0.00 (0/48) 95% *CI*: 0.00–7.41	0.00 (0/48) 95% *CI*: 0.00–7.41
Jiaxing City	3.51 (2/57) 95% *CI*: 0.62–11.92	0.00 (0/57) 95% *CI*: 0.00–6.31	0.00 (0/57) 95% *CI*: 0.00–6.31	0.00 (0/57) 95% *CI*: 0.00–6.31	0.00 (0/9) 95% *CI*: 0.00–29.91	0.00 (0/9) 95% *CI*: 0.00–29.91	0.00 (0/9) 95% *CI*: 0.00–29.91	0.00 (0/9) 95% *CI*: 0.00–29.91
Jinhua City	10.71 (9/84) 95% *CI*: 5.74–19.13	8.33 (7/84) 95% *CI*: 4.10–16.22)	0.00 (0/84) 95% *CI*: 0.00–4.37	0.00 (0/84) 95% *CI*: 0.00–4.37	8.33 (2/24) 95% *CI*: 1.48–25.85	0.00 (0/24) 95% *CI*: 0.00–13.8	0.00 (0/24) 95% *CI*: 0.00–13.8	0.00 (0/24) 95% *CI*: 0.00–13.8
Lishui City	0.00 (0/45) 95% *CI*: 0.00–7.87	11.11 (5/45) 95% *CI*: 4.84–23.50	2.22 (1/45) 95% *CI*: 0.11–11.57	11.11 (5/45) 95% *CI*: 4.84–23.50	0.00 (0/16) 95% *CI*: 0.00–19.36	0.00 (0/16) 95% *CI*: 0.00–19.36	0.00 (0/16) 95% *CI*: 0.00–19.36	0.00 (0/16) 95% *CI*: 0.00–19.36
Ningbo City	9.78 (9/92) 95% *CI*: 5.23–17.56	1.09 (1/92) 95% *CI*: 0.06–5.90	0.00 (0/92) 95% *CI*: 0.00–8.03	0.00 (0/92) 95% *CI*: 0.00–8.03	0.00 (0/50) 95% *CI*: 0.00–7.13	2.00 (1/50) 95% *CI*: 0.1–10.5	0.00 (0/50) 95% *CI*: 0.00–7.13	0.00 (0/50) 95% *CI*: 0.00–7.13
Quzhou City	9.84 (6/61) 95% *CI*: 4.59–19.85	0.00 (0/61) 95% *CI*: 0.00–5.92	0.00 (0/61) 95% *CI*: 0.00–5.92	0.00 (0/61) 95% *CI*: 0.00–5.92	0.00 (0/28) 95% *CI*: 0.00–12.06	0.00 (0/28) 95% *CI*: 0.00–12.06	0.00 (0/28) 95% *CI*: 0.00–12.06	00.00 (0/28) 95% *CI*: 0.00–12.06
Shaoxing City	0.70 (1/142) 95% *CI*: 0.04–3.88	7.04 (10/142) 95% *CI*: 3.87–12.48	0.00 (0/142) 95% *CI*: 0.00–2.63	0.00 (0/142) 95% *CI*: 0.00–2.63	0.00 (0/43) 95% *CI*: 0.00–8.20	0.00 (0/43) 95% *CI*: 0.00–8.20	0.00 (0/43) 95% *CI*: 0.00–8.20	0.00 (0/43) 95% *CI*: 0.00–8.20
Taizhou City	2.53 (2/79) 95% *CI*: 0.45–8.77	11.39 (9/79) 95% *CI*: 6.11–20.26	0.00 (0/79) 95% *CI*: 0.00–2.63	3.80 (3/79) 95% *CI*: 1.04–10.58	0.00 (0/8) 95% *CI*: 0.00–32.44	0.00 (0/8) 95% *CI*: 0.00–32.44	0.00 (0/8) 95% *CI*: 0.00–32.44	0.00 (0/8) 95% *CI*: 0.00–32.44
Wenzhou City	17.24 (20/116) 95% *CI*: 11.45–25.14	0.00 (0/116) 95% *CI*: 0.00–3.21	0.00 (0/116) 95% *CI*: 0.00–3.21	0.00 (0/116) 95% *CI*: 0.00–3.21	19.35 (6/31) 95% *CI*: 9.19–36.28	0.00 (0/31) 95% *CI*: 0.00–11.03	0.00 (0/31) 95% *CI*: 0.00–11.03	3.23 (1/31) 95% *CI*: 0.17–16.19
Zhoushan City	3.33 (2/60) 95% *CI*: 0.59–11.36	0.00 (0/60) 95% *CI*: 0.00–6.02	0.00 (0/60) 95% *CI*: 0.00–6.02	0.00 (0/60) 95% *CI*: 0.00–6.02	2.38 (1/42) 95% *CI*: 0.12–12.32	0.00 (0/42) 95% *CI*: 0.00–8.38	14.29 (6/42) 95% *CI*: 6.72–27.84	0.00 (0/42) 95% *CI*: 0.00–8.38
Zhejiang Province	5.36 (62/1156) 95% *CI*: 4.21–6.82	7.61 (88/1156) 95% *CI*: 6.22–9.29	0.17 (2/1156) 95% *CI*: 0.03–0.63	1.56 (18/156) 95% *CI*: 0.99–2.45	2.08 (17/818) 95% *CI*: 1.30–3.30	0.12 (1/818) 95% *CI*: 0.01–0.69	1.10 (9/818) 95% *CI*: 0.58–2.08	0.12 (1/818) 95% *CI*: 0.01–0.69

*CI* confidence intervals

In aspects of administrative division, the highest prevalence of *T. canis* infection was found in Wenzhou, Zhejiang (Table 1). *To. leonina* infection was detected in only two fecal samples, with one sample collected from Hangzhou and the other from Lishui, Zhejiang (Table 1). Additionally, variations in the prevalence of nematode infections were noted among cities for *A. caninum* and *Tr. vulpis* (Table 1).

### Prevalence of *Toxocara* infection in cats

Eggs of four intestinal nematode species—*T. cati*, *To. leonina*, *Tr. campanula*, and uncommonly *T. canis*—were detected in 818 cat fecal samples collected in Zhejiang Province. The prevalences of infection were estimated at 2.08% (17/818; 95% *CI:* 61.30–3.30), 1.10% (9/818; 95% *CI:* 0.58–2.09), 0.12% (1/818; 95% *CI:* 0.01–0.69) and 0.12% (1/818; 95% *CI:* 0.01–0.69), respectively. Although co-infections involving two nematode species were detected, their occurrence rate was very low. In aspects of administrative division, the highest prevalence of *T. cati* infection was found in Wenzhou, Zhejiang (Table 1; Additional information 1: Table S2).

### Risk factors for *Toxocara* infection in dogs and cats

**Fig. 1:**
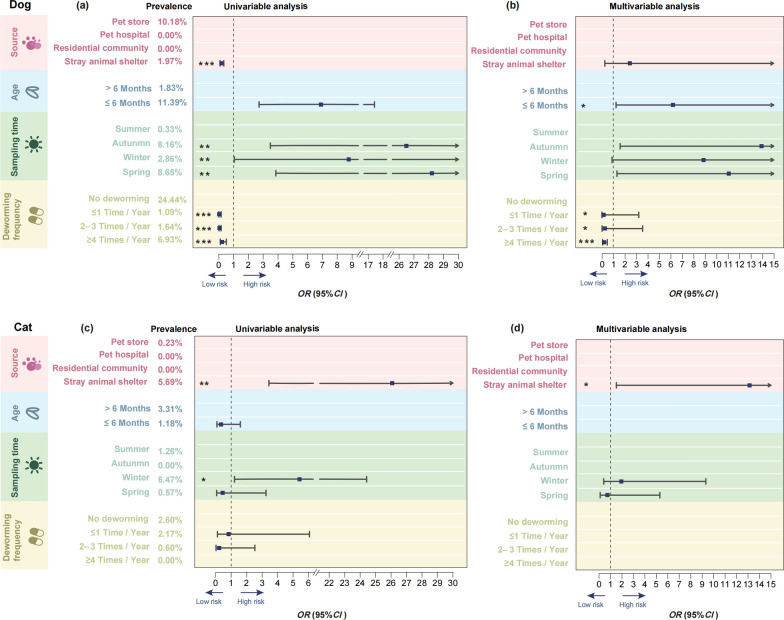
Risk factors for *Toxocara* infection in dogs and cats in Zhejiang, China. Univariable (**a**) and multivariable regression analyses (**b**) of risk factors for *T. canis* infection in dogs in Zhejiang. Univariable (**c**) and multivariable regression analyses (**d**) of risk factors for *T. cati* infection in cats in Zhejiang. Fecal sample source (pet store, pet hospital, residential community, and animal shelter), age of dog or cat (≤ 6 months and > 6 months), sampling time (spring, summer, autumn, and winter) and information form questionnaire like deworming frequency (0, 1, 2–3, or ≥ 4) and the associated detection ratio of *Toxocara* infection in the fecal samples are indicated. * indicates a *P* value < 0.05, ** indicates a *P* value < 0.01, *** indicates a *P* value < 0.001. The vertical line with a value of 1 is the Line of Null Effect. The advantage ratio (*OR*) value was deemed statistically significant if the mean with ninety-five confidence intervals (95% *CI*; horizontal lines) did not encompass a value of 1. Low and high risks are indicated by left and right arrows, respectively

Univariable logistics regression analysis revealed significant correlation between *T. canis* infection in dogs and their source, age, sampling time and deworming frequency (Fig. 1a; Additional information 1: Table S3). The prevalence of *T. canis* infection was significantly lower in dogs from stray animal shelters (1.97%; 12/608) compared to those from pet stores (10.18%; 50/491; *OR* = 0.18, *P* < 0.001). Puppies aged ≤ 6 months showed significantly higher infection rates (11.39%; 55/483) than dogs elder than 6 months old (1.83%; 5/273; *OR* = 6.89, *P* < 0.001). Seasonal peaks in infection rates were observed in spring (*OR* = 28.21, *P* = 0.001) and autumn compared to other seasons (*OR* = 26.49, *P* = 0.002). Importantly, a higher frequency of deworming was associated with a lower prevalence of *T. canis* infection in dogs (*P* < 0.001).

A multivariable logistics regression analysis was conducted by selecting variables with a significance level of *P* < 0.05 as independent variables. The analysis revealed that *T. canis* infection in dogs in Zhejiang Province was significantly correlated with “age” “sampling time” and “deworming frequency” (Fig. 1b; Additional information 1: Table S3). Dogs under six months of age (*OR* = 6.22, *P* = 0.026) and those with a deworming frequency of less than 4 times per year (*OR* = 0.18, *P* < 0.001) were more likely to be infected with *T. canis* during autumn (*OR* = 13.93,* P* = 0.017) or spring (*OR* = 11.07, *P* = 0.027).

By contrast, in cats, univariable analysis showed that a significant correlation between the infection rate of *T. cati* in Zhejiang Province and the variables “source” and “sampling time” (Fig. 1c). The probability of *T. cati* infection was higher in stray animals compared to cats from pet stores (*OR* = 26.08, *P* = 0.002), higher in winter than in summer (*OR* = 5.43, *P* = 0.028), and higher in cats with a lower frequency of deworming (Additional information 1: Table S3). Multivariable analysis showed that cats in animal shelters were more likely to exhibit a high infection rate of *T. cati* (*OR* = 13.14, *P* = 0.020) (Fig. 1d; Additional information 1: Table S3).

### Seroprevalence of human toxocariasis and risk factors

**Fig. 2:**
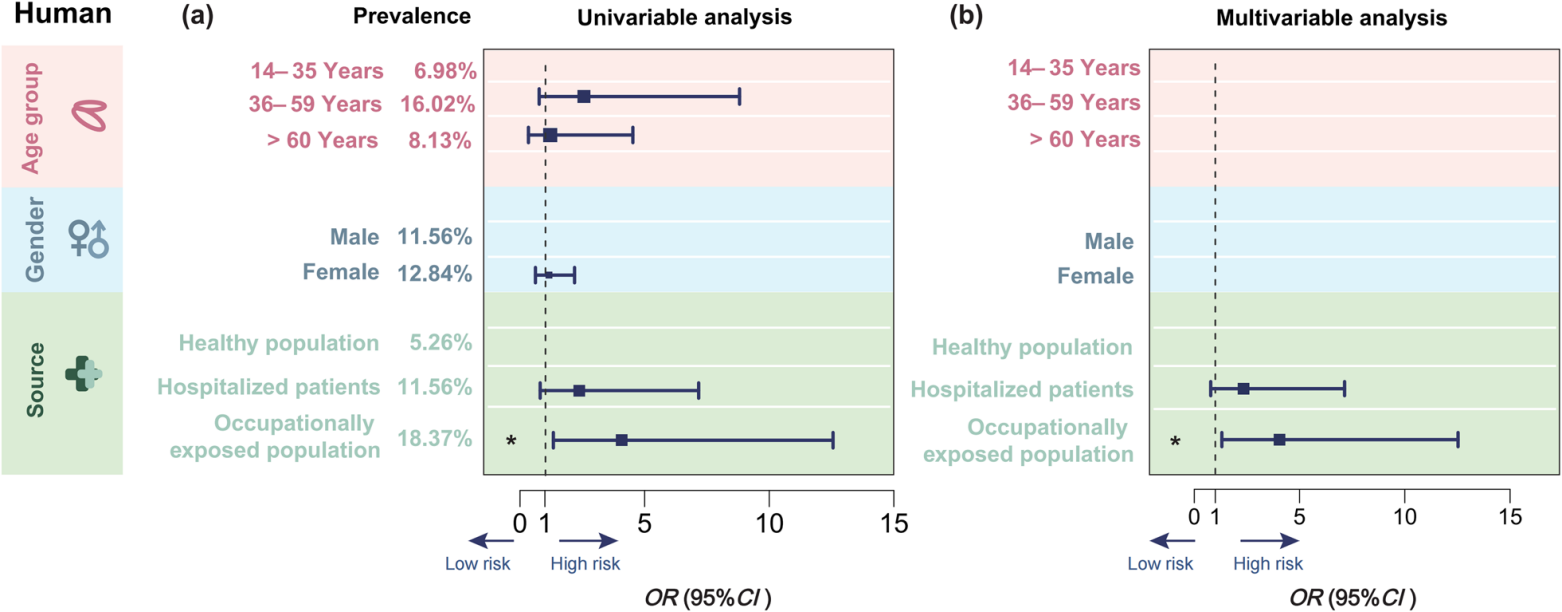
Risk factors for *Toxocara* infection/exposure in humans in Zhejiang, China. Univariable (**a**) and multivariable regression analyses (**b**) of risk factors for *Toxocara* infection or exposure in humans in Zhejiang. Age group (14–35, 36–59, or ≥ 60 years old), gender (female and male), source of serum sample (healthy, occupationally exposed or hospitalized individuals) and the associated seropositivity of anti-*Toxocara* antibodies are indicated. * indicates a *P* value < 0.05. The vertical line with a value of 1 is the Line of Null Effect. The advantage ratio (*OR*) value was deemed statistically significant if the mean with ninety-five confidence intervals (95% *CI*; horizontal lines) did not encompass a value of 1. Low and high risks are indicated by left and right arrows, respectively

A total of 347 serum samples were collected from Hangzhou (*n* = 249), Ningbo (*n* = 33), Taizhou (*n* = 32), and Zhoushan (*n* = 33) in Zhejiang Province and examined for anti-*Toxocara* IgG antibodies. The overall detection rate of anti-*Toxocara* IgG antibodies in these serum samples was 12.10% (42/347; 95% *CI:* 9.08–15.96) (Additional information 1: Table S4). A similar seropositivity of *Toxocara* infection was found between females (12.84%; 19/148; 95% *CI:* 8.38–19.18) and males (11.56%; 23/199; 95% *CI:* 7.83–16.75). No data were extracted for children. Univariable analysis indicated that a high *Toxocara* seropositivity rate (16.02%; 29/181; 95% *CI:* 11.39–22.06) was associated with middle-aged (36–59 years old) individuals (*OR* = 2.54, *P* = 0.140) and occupationally exposed populations (18.37%; 18/98; 95% *CI:* 11.95–27.18; *OR* = 4.05, *P* = 0.015) (Fig. 2a). Similar results were also obtained from the multivariable analysis (Fig. 2b). Based on these findings, age and occupational exposure represent major risk factors for human toxocariasis in Zhejiang Province.

## Discussion

Human toxocariasis is one of the top five neglected parasitic diseases that warrants for public health action [1]. It is estimated that approximately 1.4 billion people are infected with or have been exposed to *Toxocara* spp., making this one of the most neglected poverty-related infections in the world [3, 4]. However, public knowledge about human toxocariasis and its transmission route are very limited in China and most other undeveloped countries in the world. In this study, we conducted epidemiological studies to determine the prevalence of *Toxocara* infection in dogs, cats and humans in Zhejiang Province, China—the province with the most balanced urban and rural development in the nation. The aim was to raise public awareness of human toxocariasis, as a call for “One Health” intervention of this important zoonotic disease.

It was found that *T. canis* is the most prevalent zoonotic nematode in dogs in Zhejiang, China. Although the highest prevalence of nematode infection in dogs was Ancylostomatidae (e.g., *A. caninum*, confirmed by PCR amplification), it occasionally develops to adults in humans hence limited significance [33], therefore we focused particularly on *T. canis*. The prevalence of *T. canis* infection in dogs in Zhejiang (5.36%) is similar to that in Beijing (3.5%) [34] but lower than that in Guangxi (30%) [35], Hunan (45.20%) [36], and Heilongjiang (36.50%) [37]. It is also lower than the estimated overall prevalence in China (17.34%) [38] and the estimated global prevalence (11%) [39]. The different prevalence of *Toxocara* infection in dogs among provincial-level administrative divisions (PLADs) in China and other regions of the world might attribute to geographic disparities, sample sizes, differing deworming frequencies, and importantly disparate public health management contexts. Interestingly, no eggs of flatworms were detected in the fecal samples of dogs studied. It is likely a result of limited transmission, rather than a technical issue, but warrants further investigations. The risk factors of *T. canis* infection in dogs were determined as age (less than six months) and season (spring and autumn). Namely, puppies less than six months of age are more susceptible to *T. canis* infection in spring and autumn, explaining the probability of infection with *T. canis* in mostly adult dogs from stray animal shelters was lower than that of dogs mostly puppies from pet stores. This phenomenon can be attributed to the life history of *T. canis* in puppies where the parasite can complete its life cycle, and the activation of arrested larvae in female dogs and their vertical transmission to fetus [16, 20]. The high prevalence of *T. canis* infection during spring and autumn may be attributed to favorable temperatures and substantial precipitation, which provide suitable conditions for the survival and development of *T. canis* eggs in the environment. Conversely, unfavorable conditions (e.g., low or high temperatures) during other seasons may result in premature hatching and desiccation of *T. canis* eggs, potentially reducing infection rates [40]. These findings unequivocally indicate that individuals intending to raise pet dogs should be careful when cleaning puppy feces during spring and autumn. In addition, people are encouraged to adopt puppies older than six months, as they are comparatively less susceptible to *T. canis* infection. Furthermore, regular deworming of dogs is strongly recommended, regardless of whether they are raised in urban or rural areas [41–47].

Similarly, *T. cati* is the most prevalent zoonotic nematode in cats in Zhejiang, China, with an estimated prevalence of 2.08%. Although *T. canis* (dog roundworm) eggs have been found in cat feces, as previously reported [48] and molecularly confirmed in this study, this is likely due to the accidental ingestion of dog feces containing *T. canis* eggs. Specifically, *T. cati* eggs were commonly found in the feces from cats in animal shelters compared to other sources, which may be attributed to the malnutrition and long-term stress experienced by stray animals. This observation can be supported by both univariate and multivariate logistic regression analyses, which identified animal shelter a high-risk environment for *T. cati* infection in cats. Notably, the feces of stray cats often remain uncleaned, posing a continuous source of infection for cats and a potential risk for transmission to other hosts, including humans [49, 50]. Unlike the prevalence patterns observed in dogs, *Toxocara* infection in cats is more found in winter, with significant seasonal variation (*OR* = 5.43, *P* = 0.028). The fecal burial behavior of cats may provide additional protection for *T. cati* eggs in the environment during winter, thereby extending their viability and the transmission window. As the significance of *T. cati* in human toxocariasis might be underestimated [15, 29, 51], deworming and hygiene measures are also import for controlling *Toxocara* infection in cats and preventing their transmission to humans.

*Toxocara* infection or exposure is common in humans in Zhejiang, China. Our findings revealed an overall seroprevalence of anti-*Toxocara* IgG antibodies of 12.10%, which was relatively lower than that observed in a previous study in Shandong, China (12.25%) [30], as well as other studies in China (ranging from 12.14 to 44.83%) [11], and lower than the estimated global seropositivity rate (19.0%) [3]. This lower prevalence may be attributed to the balanced and adequate urban-rural development in Zhejiang (i.e., favorable economic conditions and social welfare), rather than technical limitations, as well-established sero-diagnostic assays were employed in this study [52–54]. Specifically, seropositivity for toxocariasis was predominantly found in the middle-aged group in Zhejiang, similar to findings in the United States [44]. This should be a consequent of occupational exposure, as the primary working population engaged in agriculture, farming, and slaughtering is more likely to encounter contaminated soil, pets, and wildlife, potentially leading to infection. In addition, it has been reported that antibodies to *Toxocara* spp*.* can persist for a long period of time [55], which might also explain the high prevalence of antibodies to *Toxocara* spp. in the middle-aged group. The seroprevalence rate was observed to be lower in individuals aged over 60 years, which may be related to immunosenescence [56]. Furthermore, seroprevalence was slightly higher in females than in males, consistent with findings from studies in Brazil and Shandong, China [30, 57], though not supported by other studies [47, 58]. Whether gender is a risk factor for *Toxocara* infection and whether toxocariasis is highly prevalent in children in Zhejiang warrant further investigations.

Although *Toxocara* species are globally distributed, the relatively low prevalence of these zoonotic worms in dogs, cats and humans in Zhejiang, is expected but remains underrecognized. Zhejiang is one of China’s most affluent areas, with a per capita disposable income of CNY 52,400 (USD 8106) in 2020—ranking third nationally after Shanghai and Beijing and 1.63 times the national average. Despite this balanced urban–rural development, the overall seroprevalence of human toxocariasis exceeds 12%. This underscores the importance of raising public awareness about human toxocariasis in regions with less balanced or inadequate development in China. To achieve this, nation-wide epidemiological studies focusing on middle-aged population and, preferably children should be conducted, addressing potential limitations of the current work: 1) Beyond dogs and cats, other animals can be the carriers of *Toxocara* spp., and contact with these animals, their feces, sewage, or waste represents a transmission route [17, 59]; 2) Fecal examination cannot identify latent infection in dogs, cats, or food animals harboring arrested larvae of *Toxocara* species [60, 12, 61–63]; 3) Current immunodiagnostic tools cannot distinguish between *Toxocara* infection and mere exposure in humans; 4) The absence of a “One Health (dogs/cats-environment-humans)” framework hinders effective control of human toxocariasis.

## Conclusions

The overall prevalences of *Toxocara* infection in dogs, cats, and humans were calculated at 5.36% (62/1156), 2.08% (17/818), and 12.10% (42/347), respectively, in Zhejiang Province, China. Considering the good economic status and social welfare in this province, there might be a higher prevalence of this zoonotic disease in other PLADs of China. Public awareness and knowledge about human toxocariasis and the associated risk factors (particularly pet deworming and occupational exposure) for *Toxocara* infection and transmission warrant further improvement as a “One Health” action in China.

## Supplementary Information

Additional file 1

## Data Availability

All data generated or analysed during this study are included in this article, and supplementary information.

## Abbreviations

VLM: Visceral larva migrans
OLM: Ocular larva migrans
NT: Neural toxocariasis
CDC: Center for Disease Control and Prevention
PCR: Polymerase chain reaction
*CI*: Confidence intervals
PLADs: Provincial-level administrative divisions
*OR*: Odds ratio
